# A Systematic Review of Closed-Loop Feedback Techniques in Sleep Studies—Related Issues and Future Directions

**DOI:** 10.3390/s20102770

**Published:** 2020-05-13

**Authors:** Jinyoung Choi, Moonyoung Kwon, Sung Chan Jun

**Affiliations:** School of Electrical Engineering and Computer Science, Gwangju Institute of Science and Technology, Gwangju 61005, Korea; jinyoungchoi@gist.ac.kr (J.C.); mykwon@gist.ac.kr (M.K.)

**Keywords:** EEG, closed-loop system, sleep

## Abstract

Advances in computer processing technology have enabled researchers to analyze real-time brain activity and build real-time closed-loop paradigms. In many fields, the effectiveness of these closed-loop protocols has proven to be better than that of the simple open-loop paradigms. Recently, sleep studies have attracted much attention as one possible application of closed-loop paradigms. To date, several studies that used closed-loop paradigms have been reported in the sleep-related literature and recommend a closed-loop feedback system to enhance specific brain activity during sleep, which leads to improvements in sleep’s effects, such as memory consolidation. However, to the best of our knowledge, no report has reviewed and discussed the detailed technical issues that arise in designing sleep closed-loop paradigms. In this paper, we reviewed the most recent reports on sleep closed-loop paradigms and offered an in-depth discussion of some of their technical issues. We found 148 journal articles strongly related with ‘sleep and stimulation’ and reviewed 20 articles on closed-loop feedback sleep studies. We focused on human sleep studies conducting any modality of feedback stimulation. Then we introduced the main component of the closed-loop system and summarized several open-source libraries, which are widely used in closed-loop systems, with step-by-step guidelines for closed-loop system implementation for sleep. Further, we proposed future directions for sleep research with closed-loop feedback systems, which provide some insight into closed-loop feedback systems.

## 1. Closed-Loop and Sleep Research

### 1.1. Closed-Loop Paradigms

In general stimulation experiments, stimuli are presented according to predefined stimulation parameters independent of brain activity. This is referred to as an open-loop stimulation paradigm, which is a conventional way to investigate cause-and-effect phenomena. In this case, the effect of the stimulation is commonly analyzed by comparing brain activities (induced directly) or behavior scores (induced indirectly) under various stimulation conditions. In contrast, implementation of a loop between neural circuits (e.g., human brain and data acquisition device) and external environments (such as computer, robot, or device to be controlled) is referred to as a closed-loop stimulation paradigm [1], which is among the ways to control the external environment based on neurophysiological information and to provide feedback to subjects, therefore influencing their brain activities. 

Systems that implement closed-loop paradigms have been widely used in many areas in which stimulation is applied. Studies have attempted to improve animals’ performances of tasks by measuring their brain signals to deliver electrical stimulation in real time [2,3]. In addition to electrical stimulation, closed-loop feedback systems that use optogenetics have also been proposed [4,5]. In humans, researchers have introduced some feedback systems to improve the ability to use neuroprosthetic devices [6,7,8,9], as well as closed-loop deep brain stimulation (DBS) systems, to reduce dyskinesia and paralysis caused by Parkinson’s disease [10,11,12]. Further, in the field of the brain-computer interface (BCI), closed-loop techniques are used more naturally in the form of neurofeedback to increase BCI systems’ operability [13,14,15].

### 1.2. Open-Loop and Closed-Loop Systems in Sleep Studies–Issues of Current Progress

To date, various open-loop stimulation methods have been used to elucidate sleep characteristics [16,17,18,19,20,21,22,23,24,25,26,27,28]. Among them, one study [16] revealed the mechanism and role of sleep spindle waves by applying a spike stimulus to the thalamus to induce sleep spindles. Moreover, sleep studies using transcranial direct current stimulation (tDCS) reported improved cognitive functions in attention-deficit/hyperactivity disorder (ADHD) children [17] as well as in healthy persons [18]. Saebipour et al. [19] reported a sleep stabilization effect from tDCS during insomnia patients’ sleep. Bellesi et al. [20] also attempted to reveal the mechanism of slow waves evoked by sensory stimulation and transcranial magnetic stimulation (TMS). Similarly, by applying tone stimuli to subjects during sleep, Ngo et al. reported enhanced slow wave activity (SWA) and relatively deeper sleep in sleep stages [21]. Moreover, olfactory sensory stimulation was applied in sleep studies [22,23,24], and researchers reported enhanced slow wave sleep (SWS) [22], delta activity, and spindle activity [23] as a result of olfactory stimulation. Arzi et al. [24] observed positive behavioral change (cigarette-smoking cessation) after aversive olfactory conditioning during non-rapid eye movement (NREM) sleep stage 2. Vestibular stimulation using electrical stimulation [25] and an actual rocking bed [26,27] were tested during sleep; the authors reported shortened sleep onset [25], increased amount of NREM sleep stage 2, and spindle density [26]. Omlin and colleagues also observed an increased number of spindles during the stimulation but found no effect on sleep onset or memory consolidation [27]. Lastly, tactile stimulation during sleep [28] was also introduced, and relatively greater slow oscillation (SO) density during the stimulation condition compared to the sham condition was reported; however, they found that tactile stimulation did not significantly enhance memory consolidation.

In spite of these reports, the first issue of the open-loop stimulation paradigms is that they offer only a limited ability to understand the mechanisms of sleep because it is not easy to evaluate the change caused by stimulation during sleep without affecting any other sleep parameter. For example, if one tries to investigate the role of rapid eye movement (REM) sleep on memory, one needs to modulate the REM sleep period using stimulation while controlling other parameters, and then must check the correlations between behavioral change and factors related to REM sleep. For the realization of such an idea, targeting stimulation of a specific sleep component could be achieved by introducing a closed-loop feedback system. 

The second issue is the complexity of the closed-loop system, which causes entry barriers to the sleep investigators who introduce feedback-control paradigms for sophisticated experimental design. In practice, it is not easy to introduce a commercial acquisition device on the new software platform. Thus, one is required to understand system software and modify the internal source code to connect the acquisition device into processing software. Fortunately, there are reports [29,30,31,32,33,34] about some open-source libraries widely used in closed-loop systems. They provide external libraries to connect a device easily; further, some platforms support a graphical user interface (GUI) that enables the simple introduction of a processing and feedback loop. 

### 1.3. Review Objectives

This study was designed to summarize the current progress of sleep studies with closed-loop feedback systems and provide guidelines and information on development of a closed-loop feedback system in a sleep study. To achieve these goals, we introduced the research question: “What is the main concern of sleep studies that introduce closed-loop feedback paradigms?” When dealing with this question, we tried to provide an overview of current sleep research studies that include a closed-loop feedback system.

After dealing with the systematic review of sleep studies using a closed-loop system, we will provide structural insight into closed-loop feedback systems and a list of available open-source libraries for a closed-loop system. It is expected that these contents will be useful in introducing a closed-loop feedback system in sleep studies. Currently, there are only a few articles (to the best of our knowledge, there are fewer than 10 reports) about open-source libraries for the closed-loop system, which is not good enough to perform a systematic review for this topic. Then, we will give guidelines on an implementation of closed-loop feedback systems for sleep research to minimize trial-and-error during the research process.

In the last part of this paper, we will discuss the current progress of sleep research using a closed-loop feedback system and its limitations. The future direction of sleep research and the applicability of closed-loop feedback sleep research will be suggested.

## 2. Systematic Review

### 2.1. Information Sources and Inclusion/Prescreening Criteria

In this review, the Preferred Reporting Items for Systematic reviews and Meta-Analysis (PRISMA) [35] protocol was employed (please refer the Table S1 of supplementary material for the checklist). For the literature survey, we utilized online databases: IEEE Xplore, PubMed, Web of Science, and Scopus. 

Inclusion criteria include journal articles written in English, with the exception of unpublished articles, conference proceedings, dissertations, and newspapers. Keywords used in search engines were combinations of “sleep” or “nap” with another combination of “stimul-” or “tDCS” or “tACS” (an abbreviation of transcranial alternating current stimulation), “tRCS” (an abbreviation of transcranial randomized current stimulation), “tCS” (an abbreviation of transcranial current stimulation), or “TMS” for all fields. We selected such comprehensive keywords because we noticed that even sleep studies using a closed-loop feedback system are unlikely to include keyword “closed-loop” for the title, in keywords, or even in abstracts. Therefore, we established a search strategy of finding sleep studies that included any kind of stimulation paradigms first, followed by selecting articles to meet the eligibility criteria of closed-loop feedback studies.

**Figure 1:**
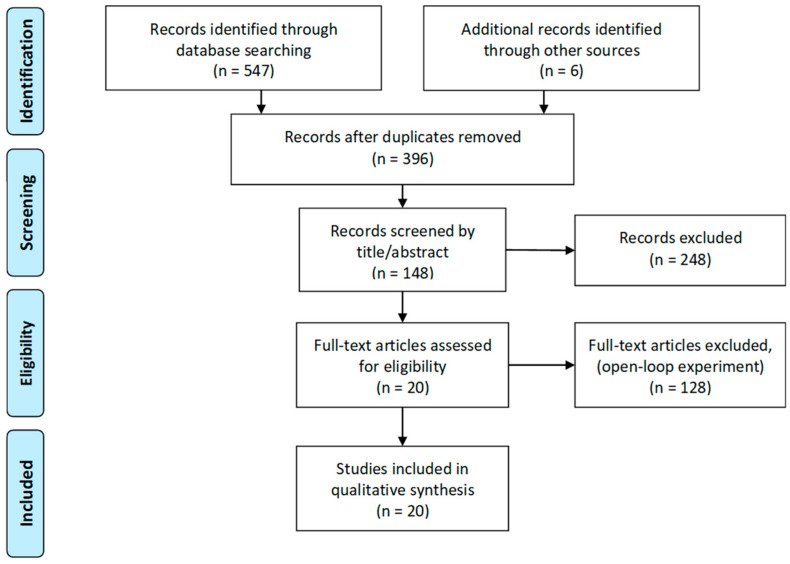
Preferred Reporting Items for Systematic reviews and Meta-Analysis (PRISMA) flow diagram of closed-loop feedback sleep studies.

Based on this search terminology, we added more options for narrowing the results in each search engine. We list the exact search terminology for each search engine in the Section from 1.1 to 1.4 of the supplementary material. Finally, 396 articles remained after duplicates were removed. We prescreened these articles for whether or not they are related to human sleep research and include stimulation protocols based on titles and abstracts. The flow diagram of this review is illustrated in Figure 1.

### 2.2. Eligibility Criteria

After prescreening, we checked the eligibility of articles through full-text screening according to the following criteria:

- Populations: Studies conducted with human subjects, but any studies with non-human subjects were excluded.
- Interventions: All types of sleep studies, including stimulation paradigms, such as transcranial electric stimulation (tES) or sensory stimulation.
- Comparators: Studies with multiple groups or a single group investigating the effect of closed-loop were considered.
- Outcomes: Studies reported regarding changed or unchanged neurophysiological/behavioral factors as a result of the experiments.
- Study designs: Studies with feedback-controlled sleep experiment design for selective stimulation were selected.

### 2.3. Search Results and Discussion

After eligibility screening, 20 studies (articles) remained for the review. In these 20 studies, we found common experimental features such as stimulation modality, target activity, and main hypotheses related to the behavioral result or mechanism of sleep.

#### 2.3.1. Stimulation Modality

**Figure 2:**
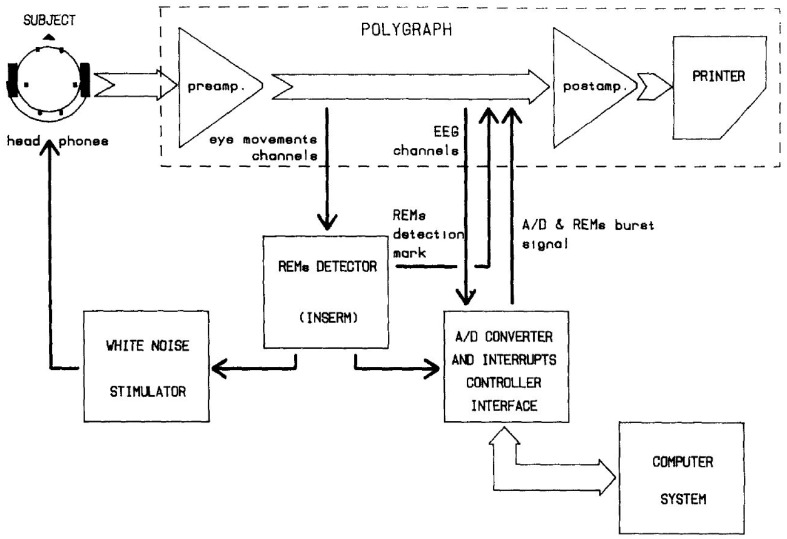
The first illustrative figure on close-loop feedback in Mouze-Amady et al. [49] This diagram demonstrates the feedback loop which is indispensable for closed-loop feedback system.

The first sleep study to introduce the closed-loop system was performed by Mouze-Amady et al. [30] in the mid-1980s. They introduced the REM detector and triggered white noise stimulation in the subject. By delivering white noise with every REM detection moment, a promoting effect on REM sleep was observed in the small-scale (four subjects) experiment. Interestingly, researchers did not address terminology such as “closed-loop”, even though the first representative figure in this article illustrated a common closed-loop feedback mechanism (Figure 2). Thereafter, closed-loop feedback studies were lacking for about three decades. In the early 2010s, Ngo et al. conducted the second sleep study that introduced the closed-loop system in an acoustic stimulation experiment that targeted slow waves during sleep [36,37]. When acoustic stimulation was delivered at the peak point of the slow wave, they observed enhanced slow wave activity that resulted in improved declarative memory consolidation. Phase-locked stimulation systems were also introduced to deliver the stimuli to consider the phase of slow oscillation [38,39,40,41]. Ong et al. [39] observed a phase-locked auditory-evoked response following spontaneous slow wave activity that resulted in better preservation of declarative memory in young adults. Using the same experimental design, older adults (60–84 years old) showed improved memory correlated with SWA and spindle activity enhancement [40]. 

Most of the studies discussed above introduced acoustic stimulation for the feedback of targeted neurophysiological activities. Meanwhile, Choi et al. introduced vibration stimulation for a feedback of heart rate monitoring during sleep [42]. To the best of our knowledge, other sensory modalities such as olfactory stimulation [22,23,43] or visual stimulation [44] have never been introduced in closed-loop feedback systems for sleep. Therefore, it is believed that there is great potential to expand the sleep research boundary to various closed-loop sensory feedback areas. 

Not only can the research be expanded using sensory stimulation, forms of transcranial electric stimulation (tES), such as TMS [45] or tACS [46], can also be introduced for the closed-loop feedback system. Because electromagnetic stimuli may promptly induce direct neurophysiological activity on the cortex [47,48], tES can be utilized to induce sleep-related brain signals so that one can control the specific activity during sleep. Thanks to the flexibility of the stimulation pattern in tES, researchers can expand the hypothesis to cover and target sleep-specific brain activities, such as sleep spindle [46].

#### 2.3.2. Target Activity

Acoustic stimulation is commonly introduced to induce K-complex, which is an evoked response for the sensory stimuli during sleep [50,51]. Because the main spectral component of K-complex is quite similar to SO (slow oscillation), the major acoustic closed-loop feedback studies try to target the SO to modulate its activity [36,37,38,39,40,41,52]. tCS also can be utilized to modulate SO during sleep, by delivering stimuli at the detection of SO as real-time feedback. Robinson et al. [53] and Ketz et al. [54] attempted to enhance SO through closed-loop tACS systems. They reported improved subjective sleep quality after closed-loop tACS [53] and improved long-term memory generalization [54]. Further, the waveform of tCS can be also used to modulate sleep spindle in addition to SO. Spindle shows the sigma frequency range (12–15 Hz) of the main spectral component, and it is well-known that sleep spindle is related closely to the sleep-specific memory consolidation [55,56,57]. Lustenberger et al. introduced spindle-like tCS to target the spindle activity during sleep, and reported enhancement in spectral power in the spindle range that was associated with improved cognitive functions [46].

Another hypothesis about SO’s orchestrating effect on the spindle activity [58,59,60] posited the contribution to memory consolidation of the temporal interconnection between SO and sleep spindle [61,62,63]. To verify this hypothesis, Choi et al. implemented a closed-loop system to present auditory stimuli that targeted sleep spindle activity [64]. They found a beneficial effect on procedural memory consolidation and a probable association between electroencephalograph (EEG) theta power and declarative memory consolidation. Meanwhile, amplitude-modulated white noise (AM-WN) was also introduced to closed-loop acoustic feedback experiments to verify whether spindle frequency activity can be modulated as a response to the amplitude-modulated sound of spindle waveform [65,66,67]. Antony et al. [65] found memory impairment after AM-WN stimulation within the spindle refractory period. On the other hand, no effect was found on memory consolidation that could be attributable to the AM-WN stimulation that targeted the up-state of SW [66,67].

#### 2.3.3. Main Hypothesis

Most studies considering SO and the targeted closed-loop feedback paradigm suggested the main hypothesis of the study as memory improvement accompanied by SO activity enhancement [36,37]. Indeed, SO and spindle are the well-known main components of sleep related to the memory consolidation mechanism [68,69,70,71]. As addressed in the previous sections, studies attempting to target SO for feedback assessed declarative memory consolidation effects through the memory tasks [36,37,38,39,40,41], and they observed that memory improvements were correlated with SO enhancement via closed-loop feedback [36,37,39,40]; however, in some cases, there were conflicting results from the studies under the same hypothesis. According to other literature [72], no improvement in declarative memory consolidation was observed after closed-loop pink noise feedback at the slow wave peak, which appears to be unmatched with the results found in [36] and [39]. Similarly, different reports on declarative memory improvement from open-loop tDCS intervention experiments exist [18,73]. These kinds of conflicting reports may be caused by uncontrolled conditions during the experiment, which may be supporting evidence of the importance of appropriate experimental design.

Procedural memory consolidation was also investigated by the closed-loop feedback on sleep spindle activities [46,64]. Lustenberger et al. reported spindle enhancement during the spindle-like tACS stimulation condition, which could be interpreted as the main reason for better memory consolidation effect, comparing to the control condition [46]. Choi et al. tried to deliver the pink noise input when the system detected sleep spindle activities, which is a different approach involving delayed acoustic stimulation after spindle detection introduced by Antony’s team. Choi et al. reported procedural memory improvement caused by acoustic stimulation following spindle activities [64], and Antony et al. [65] reported no declarative memory improvement from targeted memory reactivation (TMR) when using sound delivery around spindle activities [65]. These results may suggest the different respiratory periods of sleep spindle for different memory categories. Through the results of studies connected with the related hypothesis, one can imply a new hypothesis or infer the mechanism of a specific component.

Lastly, there are studies that demonstrate the various applications of closed-loop feedback systems for sleep research. Pilly et al. designed a novel tES paradigm for managing sophisticated TMR control during sleep [74]. Using 32 stimulation electrodes, they make 14 types of optimal tES patterns, and these were used for memory encoding and reactivation during sleep when delivery of tES coincides temporally with the up-state of SO. They found better memory retrieval for items cued during sleep than for the retrieval of non-cued items or items not encoded. It was inferred that this novel method is reliable and low-cost/ low-risk for boosting memory or behavioral therapy. 

In addition to the memory improvement effect of closed-loop feedback, Besedovsky et al. [52] reported the immune-supportive effect via acoustic stimulation; they observed a significant change in levels of cortisol and aldosterone after auditory SO stimulation, compared to sham conditions, which contributed to the delayed decreased in numbers of T and B lymphocytes in the blood. It was the first evidence suggesting a causal role for the SOs in regulating immunity; it may be inferred that closed-loop acoustic stimulation is applicable as a clinical approach in an easy-to-use and highly specific manner. 

Choi et al. used an electrocardiograph (ECG) for the closed-loop feedback [37]. They analyzed heart rate information from the subject’s ECG, then delivered vibrating stimulation with the rate of the specific percentage of heart rate on the subject’s back area. They reported that the heart-rate based closed-loop feedback had a stabilizing effect on the autonomic system during a 90-min nap. This evidence may support the idea of a therapeutic application of closed-loop vibration stimuli for cardiovascular health. 

Because of the variety and complexity of the studies above, an in-depth review literature would be helpful to demonstrate the effects of closed-loop feedback experiments. Therefore, we exhaustively surveyed the literature on sleep studies that used a closed-loop feedback system and summarized the stimulus types they used, the targets of stimulation, and their findings, as tabulated in Table 1.

## 3. Insight of Closed-Loop Systems for Sleep Study

**Figure 3:**
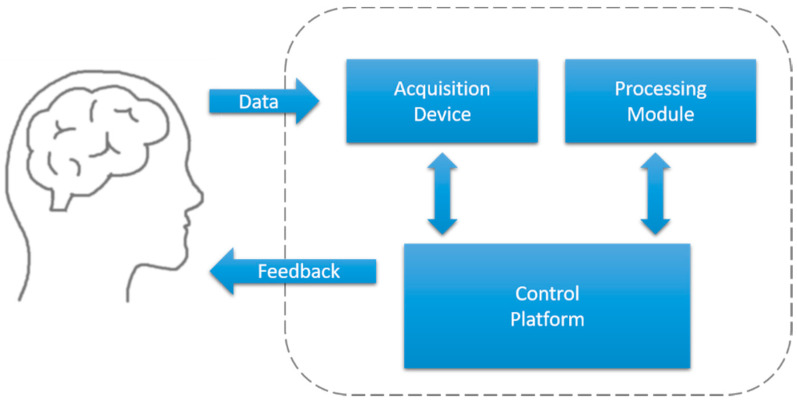
Diagram of a closed-loop feedback system.

Common closed-loop systems are composed of an acquisition device, control platform, and processing module, as illustrated in Figure 3. In this section, we describe each of these components in detail. Particularly, we focus on the closed-loop feedback system for human sleep studies using neurophysiological information such as EEG or ECG. 

### 3.1. Acquisition Device

An acquisition device refers to a device that measures analog biosignals, such as an EEG, ECG, or electromyograph (EMG). The acquisition device may convert analog biosignals (acquired from the subject’s body) into digital signals, and store or stream them. Before designing or implementing a closed-loop system, one should take into account whether one’s devices are suitable for implementing the experimental paradigm. For example, conventional EEG devices can be used in most experiments conducted in a static state (e.g., sitting on a chair). However, wireless devices are more adequate for dynamic experiments that require the subject to move. Recently, dry electrodes have been used more widely because of their convenience during the experiment; they do not require the use of gel or paste for the conductance during the experiment, and they are highly effective in hyperscanning experiments in which many subjects are involved simultaneously.

**Figure 4:**
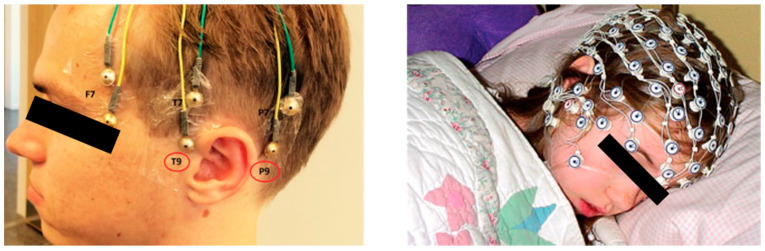
Cup-type passive electrodes (right, from Justesen et al. [76]) and mesh-style electrodes (left, Geodesic sensor net from Electrical Geodesics, Inc.).

However, in sleep studies, cup-type or mesh-style electrodes are used more commonly (Figure 4). They provide stable electrical conductance and stable attachment during nocturnal sleep or a nap. Common wet-type electrodes (e.g., active electrodes for AD-Box from BioSemi) that use a cap with conductive gel are difficult to use in sleep experiments because the subject is required to adopt a supine position. Furthermore, the viscosity of the conductive gel for this type of electrode is too low, and electrodes can shift from their proper position because the head is in contact with the pillow. Dry electrodes compatible with commercial systems are also inadequate for sleep EEG measurements because ‘headgear’-style devices, such as the DSI-24 system (Wearable Sensing), are inconvenient for a natural sleep posture, and a cap-type system with dry electrodes (g.SAHARA dry EEG electrodes from g.tec, for example) are prone to slip when an external force is applied to the cap. Thus, it is a more common practice to use cup-type electrodes with conductive paste or mesh-style electrodes in sleep EEG measurements because they remain stably adhered during sleep.

Recently, non-commercial devices for low-cost and long-term usable sleep state monitoring, as well as closed-loop feedback paradigm, were reported. Debellemaniere et al. introduced a headset-style device containing three-frontal electrodes and two mastoid electrodes [77]. They simplified the rear-part of the device by using an elastic band; therefore, they achieved the stable acquisition of data when subjects slept in supine position. They reported successful NREM sleep stage 3 detection and precise SO ascending-phase targeting stimulation via the device. Mikkelsen et al. used a dry-contact ear-EEG device for sleep monitoring, which was custom-designed for each subject [78]. Automatic sleep scoring with ear-EEG is comparable to manual scoring from conventional polysomnography (PSG) data (Cohen’s kappa of 0.73), and they showed the possibility for the usage of ear-EEG for clinical long-term sleep monitoring. Lastly, Ferster et al. designed a mobile sleep monitoring system, which measures EEG, EMG, and electrooculography (EOG) signals through electrodes placed in an elastic headband [79]. This portable device showed high accuracy when measuring correlation for delta (0.98) and sigma (0.99) frequency bands with measurement of the reference system (Embla Titanium, Embla Systems, Kanata, ON, Canada). 

These three articles emphasize the data reliability as suggesting correlation or consensus with conventional measurement. It is important to verify the quality of acquisition data because a high variability of incoming data will cause unreliable analysis results and undesirable feedback stimulation [80].

### 3.2. Control Platform

The control platform interacts with the acquisition device and processing module; it is a system-centric element that determines whether to provide feedback. For commercial devices, it is common for manufacturers to provide their proprietary software that is compatible with their devices or is device-dependent. For non-commercial or custom-made devices, one should implement one’s custom-made platform that may receive data directly from the devices and provide feedback based on the processing results. However, this work requires a full understanding of system programming, and requires considerable time and effort. Therefore, as an alternative, open-source platforms that are accessible to the public (Table 2) are widely used. Researchers should consider their compatibility with their own acquisition devices before determining the specific open-source platform. We surveyed the most popular open-source platforms and summarized their detailed information (environment supported, primary purpose, extension library language, main applications, typical system composition for the closed-loop, and so on), as tabulated in Table 2. Additionally, we note that to implement one’s experimental paradigm, options for constructing a processing module in the control platform should be considered in advance. Inherently, brain-computer interface (BCI) platforms contain components for closed-loop feedback. BCI2000 [29] and OpenViBE [30] are representative of such open-source platforms for BCI research. In BCI2000, it is possible to implement methodological algorithms to analyze brain signals in the program itself by modifying the program’s internal source code or by using various MATLAB or Python extension libraries. In OpenViBE, the scenario pipeline for signal processing can be directly set under the graphical user interface (GUI), or signal processing functions that use MATLAB or Python can be implemented easily. 

For sleep research, Choi et al. [64] used BCI2000 as a control platform to design their sleep closed-loop feedback system, and Antony et al. [65] used the OpenViBE system for the same purpose. BCILab [31] is also highly recommended and has excellent applicability in the construction of a closed-loop feedback system in the MATLAB environment. Other closed-loop systems for neuroscience also can be used for human sleep studies. For example, Lustenberger et al. introduced the RTXI system [32] for their closed-loop tACS feedback sleep study [46]. The RTXI system is commonly used for neural signal processing and closed-loop experiments with intracellular electrodes [81], but one can modify this system for real-time EEG signal processing. Lustenberger et al. used the 21-channel, whole-head EEG system, and processed sleep EEG data with the RTXI system to detect sleep spindles online. Additionally, NeuroRighter [33] was designed for experiments with micro-electrode arrays and optogenetics devices. Wu et al. [82] used this system for a closed-loop DBS experiment with mice and showed the ability to use a closed-loop application for sleep studies with closed-loop feedback. Falcon [34] is a C++-based multi-thread software that guarantees high-performance in real-time processing. While this system is designed primarily for neural signal processing, it has the potential to also be applied in sleep studies.

**Table 2:** Open-source platforms that are used widely as control platforms for a closed-loop feedback system.

Title	Environment Supported	Purpose	Extension Library Language	Applications	System Composition for Closed-Loop (ex)	URL
BCI2000 [29]	Windows	Implementing the BCI system	MATLAB, C++	Human sleep study [64], TMS for Neurorehabilitation [83], Brain-Computer Interface research [84]	Acquisition device + MATLAB signal processing + Feedback application	https://www.bci2000.org/mediawiki/index.php/Main_Page
OpenViBE [30]	Windows, Linux	Real-time brain signal processing	LUA, Python, MATLAB, C++,	Human sleep study [65], Brain-robot interface research [85], Brain-Computer Interface research [86]	Acquisition server + Python script for data processing + Feedback application	http://openvibe.inria.fr/
BCILab [31]	Windows, Linux, Mac	MATLAB toolbox for BCI research	MATLAB	Brain-machine-body interface study [87], Cognitive rehabilitation research [88]	Input plugin + Processing plugin + Output plugin	https://sccn.ucsd.edu/wiki/BCILAB
NeuroRighter [33]	Windows	A system for micro-electrode arrays and optogenetics	C#	Optogetetics [5,89], closed-loop DBS study with mice [82]	In vivo setup + NeuroRighter + Closed-loop plugin	https://sites.google.com/site/neurorighter
RTXI [32]	Linux	Real-time neural signal processing	MATLAB, C++	Human sleep study [46], Dynamic clamp [81]	Data acquisition card + Real-time(RT) code + User Interface	http://www.rtxi.org/
Falcon [34]	Linux	Population neural signal en(de)coding	Python, C++	Real-time spike pattern identification [90]	Data Sources + Processing nodes + Feedback output	https://bitbucket.org/kloostermannerflab

For some platforms, if one’s acquisition device is not on the list of compatible devices, extension libraries that allow one to link any devices to the platform may be provided without charge. However, if one is unfamiliar with this task, choosing a control platform with which one’s device is compatible is likely to be simpler and save time and effort.

### 3.3. Processing Module

The processing module is a system element used to analyze brain signals transmitted from the acquisition device. At this stage, the brain-state dynamics loop (a loop formed between system and subject based on neurophysiological information) is implemented by analyzing brain signals in real-time and yields the resulting feedback that affects neurophysiological activity [91]. It is understood that the brain signals acquired by the acquisition devices are the result of overlapping electrical activities from neurons in various brain areas [92]. The goal of the processing module is to extract meaningful information from these brain signals. Their analysis of EEG signals, in particular, is performed in the time, frequency, phase, or time-frequency domain. The detection of feature components is possible only when distinct activity is noted—i.e., when more significant characteristics than regular background EEG activity are observed. For example, an evoked response to stimulation, such as event-related potential (ERP), is observed more typically in time domain analysis because its amplitude is greater than regular background EEG activity [93]. 

In sleep EEG data, slow waves and sleep spindles are distinct and commonly observed [94]. Slow waves are known to be the main component of EEG during NREM sleep stage 3, which yields EEG with a relatively low frequency (1–4 Hz) and large amplitude. Further, sleep spindles are another important feature of NREM sleep stage 2 with a wave with a waxing and waning shape in the sigma frequency range (12–15 Hz). To elucidate the role and mechanism of these brain signals’ characteristics, many studies have implemented closed-loop systems to strengthen certain specific characteristics of brain signals as a feedback target and examine whether related brain functions are associated [36,46,95].

In addition to various distinctive components of brain activity, changes in the human mental state or variations in consciousness may cause changes in the background components of brain waves or connectivity between brain regions [96]. For example, the brain state during which no task is performed is referred to as the ‘resting-state.’ It is understood that brain connectivity may be identified in a resting-state, and changes in its connection may occur when a specific brain activity emerges [97]. As such, it is possible to implement a methodology that identifies the brain state and provides feedback based on it, rather than detecting specific brain activity.

### 3.4. Guideline for Preparing Closed-Loop Feedback Sleep Research

In this section, we introduce the process of implementing a closed-loop system for sleep research. The following is a detailed, step-by-step description of the implementation, which is based on information from a systematic review on closed-loop feedback sleep experiments.

#### 3.4.1. Hypothesis Setup

First, researchers set the target hypothesis to be verified. The advantage of the closed-loop system is that it is possible to test hypotheses about the general neurophysiological activity under strict control conditions. In Section 2.3, we found common categories that were considered in closed-loop sleep research: stimulation modality, a target for stimulation, and the main hypothesis. The stimulation modality and target should be set reasonably based on the main hypothesis of the research. Therefore, one should establish a hypothesis based on the existing literature information. 

#### 3.4.2. Selection of Acquisition Device

Commercial devices are available with various types of electrodes provided by the company. As addressed in Section 3.1, to perform a sleep EEG study, devices using passive electrodes or highly elastic mesh-type electrodes are recommended to ensure that the electrodes remain securely attached during sleep. Under magnetic field conditions such as a magnetic resonance imaging (MRI) system, electrodes made of a nonmetallic conductive material should be used. Non-commercial devices for sleep monitoring may be used for a closed-loop sleep experiment when the signal quality of the devices is verified to be comparable to that of conventional devices [77,78,79,80].

#### 3.4.3. Selection of Control Platform

For choosing the control platform, device compatibility and options for implementing processing module should be considered. Connecting a third-party device to the platform sometimes requires a full understanding of system programming, and a restrictive implementation method for processing modules may require tremendous time and effort. To the best of our knowledge, BCI2000 and OpenViBE are the typical control platforms that can be utilized for a closed-loop feedback system, and both are specialized for the development of BCI systems. They are designed to facilitate real-time EEG analysis and present users with the results of the analysis as feedback. We tabulated the list of open-source platforms (Table 2) that are considerable for closed-loop system implementation. It may be helpful in finding the best option under the current environment. 

#### 3.4.4. Implementation of the Processing Module

Next, we need to investigate options to implement real-time brain state analysis on the control platform chosen. In general, each platform includes functions to analyze EEG information and to provide feedback based on the analysis results. For instance, MATLAB signal processing libraries from BCI2000 or GUI pipeline from OpenViBE exist. Meanwhile, in some cases, a researcher should construct one’s own (purpose-specific) algorithms to apply much more sophisticated signal processing or detection algorithms to establish an experimental paradigm. If possible, depending on the study’s purpose, one may implement custom-made signal processing tools by introducing external libraries using a plugin such as EEGLab or Fieldtrip libraries [98,99].

An additional consideration in implementing the processing algorithm is EEG components’ inter-subject variability. For example, sleep spindles are generally known to manifest as EEG activity in the 11–15 Hz band; however, Cox et al. [100] reported that some subjects had a slightly different spindle frequency range. It is essential to implement a pre-processing method to consider and overcome such inter-subject variability and set the parameters of the detection algorithm according to subject-specific EEG characteristics. 

#### 3.4.5. Stimulation Parameter Adjustment (Optimization)

After the implementation of the processing module is complete, it is common to encounter unexpected problems during the experiment. To minimize such problems and to confirm system stability, it is essential to conduct several pilot tests before the full-scale experiment. During these pilot tests, algorithm-specific parameters may be explored to achieve the stable operation of the closed-loop system. In the worst case, modification of the experimental paradigm may be required to resolve a module’s critical processing issue, which may take more time and effort than expected.

#### 3.4.6. Hypothesis Verification: Analyzing Empirical Data

Once the algorithm and various critical parameters for the stable feedback loop are established, it is possible to check whether the target stimulus detection and the stimulation method are working appropriately. To do so, one may check the existence of typical responses in the experimental data and confirm whether there is any evidence to support one’s hypothesis. 

In addition to comparing the differences in responses to conditions through stimuli, it is also essential to ensure that the various dependent variables involved in the experiment are controlled well, and to confirm whether uncontrolled factors may influence the experimental results. Unsuitable stimuli will likely lead to unintended effects, such as shortening sleep, which may lead to false judgments of the consequences or may cause quite unexpected outcomes incongruent to existing evidence.

## 4. Discussion

### 4.1. Why Closed-Loop?

In this systematic review, we found 148 sleep studies with stimulating intervention. Among them, 128 studies used open-loop paradigms, and 20 studies used closed-loop feedback paradigms. Such a tremendous number of open-loop stimulation experiments, however, is limited in its ability to investigate mechanisms or to verify hypotheses about sleep-related components. A common strategy of experimentation with the open-loop stimulation paradigm is to compare the factors related to the hypothesis between different sleep conditions (stimulation vs. control). Stimulation parameters in the open-loop paradigm are set in common practice by introducing information from other literature; however, such pre-defined interventions may limit the capacity to control other factors (which may be irrelevant to the hypothesis) and affect the final result of the experiment. Despite such limitations, we found that a huge amount of open-loop research has been conducted to verify hypotheses about the mechanism or the role of sleep components like SO or sleep spindle.

Since the early 2010s, research using the closed-loop paradigm to investigate the mechanism of SO and sleep spindle has been conducted. Therefore, the contribution of SO and sleep spindle on memory consolidation was comprehended well with the feedback control technique for selective modulation of SO or spindles [36,37,38,39,40,41,75]. Furthermore, in addition to the role of memory consolidation, immune-supportive function [52] or subjective sleep quality [53] were also revealed to be related to slow wave activity by using electrical stimulation. Moreover, applying the TMR technique to the closed-loop feedback system showed the possibility and reliability of memory encoding and memory improvement techniques [65,74]. 

In these circumstances, we can understand the ripple effect of closed-loop systems, which contributes to the advancement of studies about sleep and memory. Nonetheless, there is great potential to improve the reliability of closed-loop feedback because system applicability in a real-world environment is hard to guarantee, especially for systems based on human neurophysiology. As an example, it has been a half-century since the emergence of the BCI paradigm [101,102], but there is still continuous devotion to improve the reliability of the BCI system in a practical manner [103,104]. Similarly, stimulation methods for sleep modulation also need to improve their reliability for popular use. Electrical stimulation such as tCS or TMS can be advanced by introducing parameter optimization methods through high-performance computing resources, and it is expected to guarantee the reliable sleep modulating effect [105,106]. Moreover, the adverse effect of open-loop deep brain stimulation intervention without considering the individual differences in neurophysiological characteristics was reported [107,108]. Therefore, stimulation studies should consider the closed-loop feedback technique along with parameter optimization schemes considering inter-subject variability.

Lastly, various stimulations, such as visual [44], tactile [28], and olfactory stimulation [22,23,24], can be introduced as substitutes for acoustic stimuli [36,37,38,39,40,41,52,75] for existing closed-loop studies. This feature of the closed-loop system shows the feasibility of closed-loop research for multi-disciplinary purposes. For example, Antony et al. introduced TMR, which used tone stimulation for closed-loop feedback and verified spindle refractory period on declarative memory consolidation [65]. Pilly et al. also suggested a novel memory encoding method using spatiotemporal patterns of tES and introduced TMR for cueing memory with closed-loop tES [74]. They confirmed a reliable effect of memory encoding and cueing during wake and sleep, and they suggested the possibility of a closed-loop tES system for the application of behavioral therapy or as a low-risk non-invasive approach for boosting learning and memory. Moreover, there are some reports about subjective sleep quality improvement [53], autonomic nervous stabilization [42], and improvement of immune-supportive function [52] using closed-loop feedback paradigms. Therefore, further expansion of closed-loop feedback applications in various disciplines is expected, especially for sleep-related wellness. 

### 4.2. Future Directions in Sleep Research Using a Closed-Loop System

The current closed-loop stimulation methods can only modulate some of the sleep-dependent brain activities, but researchers have continued to examine the possibility of sleep modulation [109,110]. To investigate the effect of stimulation on sleep modulation, we need to trace the sleep stages because sleep progress follows the pattern of sequences of sleep stages [111]. Common sleep staging techniques have mainly been performed by manual inspection of scorers [94]. Meanwhile, various automated sleep stage classification methods have emerged [112,113,114,115,116], and there is even artificial intelligence (AI) supported by cloud services for real-time automatic sleep scoring [114]. Therefore, it is expected that the advances in automated sleep scoring techniques would provide a sleep cycle monitoring scheme to the closed-loop feedback system, thus elevating its potential as an application for sleep modulation research.

In addition to automatic sleep stage classification, optimization of closed-loop stimulation may also be conducted by machine intelligence. For instance, Kulkarni et al. suggested a deep learning-based sleep spindle detector, which shows machine learning’s potential in designing the automated detection paradigm [117]. They reported successful generalization across sleep spindle datasets so that they achieved stable spindle detection for various subject groups with different ages and races. Such generalization or optimization of automated feedback is of great importance for the closed-loop system based on human neurophysiology. There is a report about inter-subject variability in spindle frequency and topography among subjects [100], and another report mentioned auditory-evocation potential’s varying characteristics over subjects during sleep [64]. If feedback is presented as a global parameter without considering individual differences, stimulus effects may be reduced or even yield adverse effects attributable to phase differences between spontaneous EEG activity and evoked potential [2]. Therefore, it is essential to implement a technique to determine the intensity, frequency, and phase of the stimulus based on individual EEG characteristics. Nonetheless, parameters for stimulation are manually adjusted based on the adaptive sleep EEG data to reflect such variability in EEG characteristics generally.

Future closed-loop systems will achieve full automation by the inevitable adoption of the latest machine learning techniques. We expect that a closed-loop feedback system with monitoring scheme for the sleep cycle would be effective in modulating or managing sleep. Moreover, a machine intelligence-based optimal feedback method could also contribute to sleep modulation research. These techniques could be applied to higher-level research, such as sleep optimization to improve sleep effects or quality. Nevertheless, implementation of evaluation methods should be accompanied by a modulation study to examine the effect of an intervention on any kind of concerned factors. It is common to examine cognitive tasks (e.g., working memory tasks) to assess the correlation between neurophysiological change and performance variation [118,119,120]. However, there is no clear index for sleep quality assessment except questionnaires as a subjective measure [121] or an analysis method of sleep-dependent neurophysiological characteristics (e.g., spectral power analysis for SO or sleep spindle). Therefore, an objective index for assessing sleep quality should be investigated while conducting closed-loop feedback sleep research.

## 5. Summary and Conclusions

In this work, we reviewed sleep studies that utilized closed-loop feedback paradigms. Among 20 studies, we found common keywords for categorization relating to stimulation modality, feedback target, and the main hypothesis of the study. During the systematic review, we found great advancement in SO and spindle-targeted feedback experiments since the early 2010s, and there is great potential to expand sleep research with closed-loop feedback systems under the consideration of various stimulation modalities. 

We also described the concept of a closed-loop system and the detailed considerations for implementing its components for sleep research. For the system implementation procedure, we recommended choosing a control platform that is compatible with one’s acquisition devices and checking the existence of proper functions or libraries for the implementation of the processing module. Further, we demonstrated the future direction of closed-loop feedback research for sleep. The current advancements of closed-loop paradigms may expand the applicability of the research outcomes to various areas. Additionally, it is expected that machine intelligence will be introduced for parameter optimization and sleep stage monitoring for the closed-loop feedback system. Thus, it is believed that the future direction of closed-loop sleep research will pursue sleep optimization methods, including sleep cycle modulation through fully-automated feedback techniques.

In conclusion, to date, the closed-loop system has shown great potential for the advancement of sleep research. A further advance of closed-loop feedback sleep research accompanied by the technical support of machine intelligence is expected. The list of open-source libraries and the guidelines for the implementation of a closed-loop feedback system in this work would be useful for introducing a feedback-controlled paradigm for sleep experiments. 

## Figures and Tables

**Table 1:** Sleep studies with closed-loop feedback systems.

Literature	Stimulus Type	Control Parameters	Behavioral Effects
Mouze-Amady et al. [49]	Acoustic	White noise stimulation at REM detection	Prolonged REM duration
Ngo et al. [36,37]	Acoustic	Pink noise at up-state SW	Declarative memory improvement
Besedovsky et al. [52]	Acoustic	Pink noise at up-state SW	Regulating immune-supportive function
Santostasi et al. [38] Ong et al. [39,75] Papalambros et al. [40] Leminen et al. [41]	Acoustic	Phase-locked pink noise on SO	Declarative memory improvement
Bergmann et al. [45]	TMS	TMS at up-state SW	-
Lustenberger et al. [46]	tACS	spindle-like tACS at spindle activity	Procedural memory improvement
Henin et al. [72]	Acoustic	Pink noise at up-state SW	-
Choi et al. [64]	Acoustic	Pink noise at spindle activity	Procedural memory improvement
Robinson et al. [53]	tACS	SW-like tACS in phase with SW oscillations	Improved subjective sleep quality
Ketz et al. [54]	tACS	SW-like tACS in phase with SW oscillations	Improved long-term memory generalization
Pilly et al. [74]	tDCS & tACS	TMR at up-state SW	Targeted memory improvement
Choi et al. [42]	Vibration	Vibration stimuli with a relative heart rate change	Stabilized the autonomic nervous system
Antony et al. [65]	Acoustic	TMR after spindle activity	Improved declarative memory
Ngo et al. [66]	Acoustic	Spindle-frequency AM-WN stimulation at up-state SW	-
Fattinger et al. [67]	Acoustic	Spindle-frequency AM-WN stimulation at up-state SW	-

## Supplementary Materials

The following are available online at https://www.mdpi.com/1424-8220/20/10/2770/s1, Table S1: PRISMA 2009 checklist.
