# Supplementary material for: A Systematic Review of Closed-Loop Feedback Techniques in Sleep Studies—Related Issues and Future Directions
Source: Sensors (Basel). 2020 May 13;20(10):2770. doi: 10.3390/s20102770 (PMC7285770; doi:10.3390/s20102770)
Supplement: Supplementary file 1 [file sensors-20-02770-s001.pdf]

sensors

MDPI

Review

A Systematic Review of Closed-Loop Feedback Techniques in Sleep Studies—Related Issues and Future Directions

Jinyoung Choi, Moonyoung Kwon and Sung Chan Jun *

School of Electrical Engineering and Computer Science, Gwangju Institute of Science and Technology, Gwangju 61005, Korea; jinyoungchoi@gist.ac.kr (J.C.); mykwon@gist.ac.kr (M.K.)
* Correspondence: scjun@gist.ac.kr; Tel.: +82-62-715-2216

Received: 3 March 2020; Accepted: 10 May 2020; Published: 13 May 2020

1. Searching terminologies for the article search

Here, we share the searching terminologies used for each search engine. We used common keywords, including "Sleep", "Nap", "Stimul-" (i.e., we considered the variants like "stimulation" or "stimuli") and various abbreviations of "transcranial current stimulation" (tCS) such as "tDCS", "tACS" or "tRNS".

1.1. IEEE Xplore

In the advanced search page of the IEEE Xplore website, we generated search command as below:

(("All Metadata":"Sleep" OR "All Metadata":"Nap") AND ("All Metadata":"Stimulation" OR "All Metadata":"Stimuli" OR "All Metadata":"tDCS" OR "All Metadata":"tCS" OR "All Metadata":"tACS" OR "All Metadata":"TMS"))

Using this search command, we found 40 journal articles as a result. One can use this command in the "command search" page of the advanced search to repeat the search result.

1.2. PubMed

Using the PubMed advanced search builder, we generated search syntax as below:

((((Sleep[Title]) OR Nap[Title])) AND (((((((Stimulation[Title]) OR Stimuli[Title]) OR tCS[Title]) OR tDCS[Title]) OR tACS[Title]) OR tRNS[Title]) OR TMS)) AND ((((EEG[Title/Abstract]) OR MEG[Title/Abstract]) OR MRI[Title/Abstract]) OR NIRS[Title/Abstract])

To narrow the search results, we added terminologies related to the acquisition modality such as EEG or MRI. As a result of the search, we found 148 journal articles.

1.3. Scopus

Using the advanced search of the Scopus search engine and refine tool, we generated query string as below:

(TITLE (sleep OR nap) AND TITLE (stimul* OR tcs OR tdcs OR tacs OR trns OR tms)) AND TITLE-ABS-KEY (eeg OR meg OR mri OR nirs OR electroencephalo* OR magentoencephalo* OR magnetic OR infra-red OR infra) AND (LIMIT-TO (PUBSTAGE , "final")) AND (LIMIT-TO (DOCTYPE , "ar")) AND (LIMIT-TO (LANGUAGE , "English")) AND (LIMIT-TO (SRCTYPE , "j")) AND (EXCLUDE (SUBJAREA , "BIOC") OR EXCLUDE (SUBJAREA , "PHAR") OR EXCLUDE (SUBJAREA , "AGRI"))

To exclude studies not related to sleep research with sensory or electrical stimulation, we refined data by excluding the records of the subject area belonging to "Biochemistry, Genetics and Molecular

Biology", "Pharmacology, Toxicology and Pharmaceutics", and "Agricultural and Biological Sciences". As a result, we found 284 document results.

1.4. Web of Science

From the advanced search page of Web of Science, we generated a query as below:

((TI=(sleep or nap) AND TI=(stimul* or tCS or tDCS or tACS or tRNS or TMS)) AND TI=(EEG or MEG or MRI or NIRS or electroencephalo* or magnetoencephalo* or Magnetic or infra-red or infra)) Refined by: LANGUAGES: (ENGLISH) AND DOCUMENT TYPES: (ARTICLE)

As shown in the last part of the query, we refined results by excluding meeting abstracts, editorial materials, review, proceeding papers and articles not written in English. Finally, we found 80 articles from the search.

Table S1. PRISMA 2009 Checklist [1].

Section/topic	#	Checklist item	Reported on page #
		**TITLE**	
Title	1	Identify the report as a systematic review, meta-analysis, or both.	1
		**ABSTRACT**	
Structured summary	2	Provide a structured summary including, as applicable: background; objectives; data sources; study eligibility criteria, participants, and interventions; study appraisal and synthesis methods; results; limitations; conclusions and implications of key findings; systematic review registration number.	1
		**INTRODUCTION**	
Rationale	3	Describe the rationale for the review in the context of what is already known.	2-3
Objectives	4	Provide an explicit statement of questions being addressed with reference to participants, interventions, comparisons, outcomes, and study design (PICOS).	3
		**METHODS**	
Protocol and registration	5	Indicate if a review protocol exists, if and where it can be accessed (e.g., Web address), and, if available, provide registration information including registration number.	3
Eligibility criteria	6	Specify study characteristics (e.g., PICOS, length of follow-up) and report characteristics (e.g., years considered, language, publication status) used as criteria for eligibility, giving rationale.	3
Information sources	7	Describe all information sources (e.g., databases with dates of coverage, contact with study authors to identify additional studies) in the search and date last searched.	3
Search	8	Present full electronic search strategy for at least one database, including any limits used, such that it could be repeated.	3 and section 1 of supplementary file
Study selection	9	State the process for selecting studies (i.e., screening, eligibility, included in systematic review, and, if applicable, included in the meta-analysis).	3
Data collection process	10	Describe method of data extraction from reports (e.g., piloted forms, independently, in duplicate) and any processes for obtaining and confirming data from investigators.	3
Data items	11	List and define all variables for which data were sought (e.g., PICOS, funding sources) and any assumptions and simplifications made.	3
Risk of bias in individual studies	12	Describe methods used for assessing risk of bias of individual studies (including specification of whether this was done at the study or outcome level), and how this information is to be used in any data synthesis.	Not applicable
Summary measures	13	State the principal summary measures (e.g., risk ratio, difference in means).	Not applicable

Synthesis of results	14	Describe the methods of handling data and combining results of studies, if done, including measures of consistency (e.g., I^2) for each meta-analysis.	Not applicable
Risk of bias across studies	15	Specify any assessment of risk of bias that may affect the cumulative evidence (e.g., publication bias, selective reporting within studies).	Not applicable
Additional analyses	16	Describe methods of additional analyses (e.g., sensitivity or subgroup analyses, meta-regression), if done, indicating which were pre-specified.	Not applicable
		**RESULTS**	
Study selection	17	Give numbers of studies screened, assessed for eligibility, and included in the review, with reasons for exclusions at each stage, ideally with a flow diagram.	3-4
Study characteristics	18	For each study, present characteristics for which data were extracted (e.g., study size, PICOS, follow-up period) and provide the citations.	4-7
Risk of bias within studies	19	Present data on risk of bias of each study and, if available, any outcome level assessment (see item 12).	Not applicable
Results of individual studies	20	For all outcomes considered (benefits or harms), present, for each study: (a) simple summary data for each intervention group (b) effect estimates and confidence intervals, ideally with a forest plot.	4-7
Synthesis of results	21	Present results of each meta-analysis done, including confidence intervals and measures of consistency.	Not applicable
Risk of bias across studies	22	Present results of any assessment of risk of bias across studies (see Item 15).	Not applicable
Additional analysis	23	Give results of additional analyses, if done (e.g., sensitivity or subgroup analyses, meta-regression [see Item 16]).	Not applicable
		**DISCUSSION**	
Summary of evidence	24	Summarize the main findings including the strength of evidence for each main outcome; consider their relevance to key groups (e.g., healthcare providers, users, and policy makers).	14-15
Limitations	25	Discuss limitations at study and outcome level (e.g., risk of bias), and at review-level (e.g., incomplete retrieval of identified research, reporting bias).	14-15
Conclusions	26	Provide a general interpretation of the results in the context of other evidence, and implications for future research.	14-15
		**FUNDING**	
Funding	27	Describe sources of funding for the systematic review and other support (e.g., supply of data); role of funders for the systematic review.	Not applicable

References

1. Moher D.; Liberati A.; Tetzlaff J.; Altman DG. The PRISMA Group. Preferred Reporting Items for Systematic Reviews and Meta-Analyses: The PRISMA Statement. *PLoS Med* **2009** *6*, e1000097. doi:10.1371/journal.pmed1000097.
